# A Novel 3D Label-Free Monitoring System of hES-Derived Cardiomyocyte Clusters: A Step Forward to *In Vitro* Cardiotoxicity Testing

**DOI:** 10.1371/journal.pone.0068971

**Published:** 2013-07-08

**Authors:** Heinz-Georg Jahnke, Daniella Steel, Stephan Fleischer, Diana Seidel, Randy Kurz, Silvia Vinz, Kerstin Dahlenborg, Peter Sartipy, Andrea A. Robitzki

**Affiliations:** 1 Center for Biotechnology and Biomedicine (BBZ), Molecular Biological-Biochemical Processing Tecnology, Leipzig, Germany; 2 Cellectis Stem Cells, Cellartis AB, Göteborg, Sweden; University of Cincinnati, United States of America

## Abstract

Unexpected adverse effects on the cardiovascular system remain a major challenge in the development of novel active pharmaceutical ingredients (API). To overcome the current limitations of animal-based *in vitro* and in vivo test systems, stem cell derived human cardiomyocyte clusters (hCMC) offer the opportunity for highly predictable pre-clinical testing. The three-dimensional structure of hCMC appears more representative of tissue milieu than traditional monolayer cell culture. However, there is a lack of long-term, real time monitoring systems for tissue-like cardiac material. To address this issue, we have developed a microcavity array (MCA)-based label-free monitoring system that eliminates the need for critical hCMC adhesion and outgrowth steps. In contrast, feasible field potential derived action potential recording is possible immediately after positioning within the microcavity. Moreover, this approach allows extended observation of adverse effects on hCMC. For the first time, we describe herein the monitoring of hCMC over 35 days while preserving the hCMC structure and electrophysiological characteristics. Furthermore, we demonstrated the sensitive detection and quantification of adverse API effects using E4031, doxorubicin, and noradrenaline directly on unaltered 3D cultures. The MCA system provides multi-parameter analysis capabilities incorporating field potential recording, impedance spectroscopy, and optical read-outs on individual clusters giving a comprehensive insight into induced cellular alterations within a complex cardiac culture over days or even weeks.

## Introduction

Enormous efforts and investments are made throughout the process of API development to identify adverse effects and minimize the risks for patients. The pharmaceutical industry has an urgent need for assays that are applicable at an early stage in the process of API development which, at the same time, provide highly predictive and detailed information for the human *in vivo* situation [1]. This is particularly relevant for the assessment of arrhythmia caused by new chemical entities. Current guidelines from regulatory authorities including the European Medicines Agency (EMA 2005, ICH) and the US Food and Drug Administration (FDA 2005, S7B) recommend extensive cardiotoxicity testing prior to clinical trials.

The majority of drug-induced adverse events in humans involve a delay in repolarization of the cardiac action potential and therefore, an action potential duration (APD) prolongation. This is correlated with the prolongation of the clinical relevant QT interval indicating delayed ventricular repolarization and is routinely used as a biomarker of life-threatening ventricular arrhythmia e.g. Torsades de Pointes (TdP). Testing strategies generally include basic hERG assays from single cardiac cells, such as rabbit purkinje fiber cells, through to complex *in vivo* models. Although hERG assays are relatively quick and inexpensive, the repolarization event can be affected by a multitude of ion channels and thus the simple hERG assays only provide limited information [2]. Furthermore, specific cardiotoxicity caused by e.g. cancer drugs especially in the context of chronic treatment is getting into focus of drug safety research [3]. While primary cardiomyocyte cells derived from animals have the advantage of allowing the concerted action of many ion channels involved in the action potential as well as structural analysis, interspecies differences in ion channel composition and signal cascades can hinder predictivity when extrapolating from non-human experimental models to the clinical setting [1], [4].

Therefore, the tremendous progress in the field of stem cell biology has opened the access to a limitless source of non-transformed human cells and allows the *in vitro* generation of highly purified human cardiomyocytes [5]–[7]. This advances *in vitro* assays a step closer to an improved safety assessment of novel API and the replacement of partially insufficient animal trials [1], [4]. Extensive molecular, structural, and functional studies of stem cell-derived cardiomyocytes show concordance with the cardiac phenotype and similarity to their human *ex vivo* counterparts [5], [8]–[11].

Since the three-dimensional structure plays a crucial role both during cardiomyocyte differentiation and in the pharmacologically induced responses of matured cardiomyocytes [12]–[14], the demand for suitable analytic methods for hCMC is increased. Monitoring and analysis of the whole functionality of cardiomyocytes and hCMC can be performed by manual patch clamp technique, but is limited in throughput, parallelization and is not suitable for long-term monitoring of whole hCMC over days or weeks. Microelectrode array (MEA)-based field potential recording enables a less laborious method that can be easily parallelized and upscaled [1], [15]–[17]. In addition, by using the same MEA platform, cytotoxic processes as well as alterations in cell-cell contacts can be monitored in a non-invasive and label-free manner by impedance spectroscopy [18]–[20]. This technique matches the demands on bioelectronic hCMC monitoring since it permits the quantitation of cellular alterations in complex tissues and cultures [21], [22]. Unfortunately, the commonly used planar MEA require plating and outgrowth of the precursor cluster (EB) [17] or the differentiated hCMC [16]. Although, in the latter case the benefit of hCMC-based short-term safety testing could be impressively demonstrated, the stable attachment is related to the high percentage of non-cardiomyocyte cells within the used clusters (∼ 75%). This raises concerns to the standardization and the reliability and sensitivity to test compounds since these parameters are considerable dependent on the cluster/cell composition. In this study we could demonstrate if highly purified hCMC are used the attachment on planar MEA is problematic.

To overcome this limitation, here we present a unique MCA-based system that greatly facilitates bioelectronic monitoring of three-dimensional cardiomyocyte cultures. In combination with embryonic stem cell-derived hCMC, which provide a 3D model with higher degree of cell to cell coupling than a cell monolayer, this is an interesting system for studies of multicellular events, such as for wave propagation or complex tachycardia. In this study of the multi-parametric analysis of hCMC functionality and cytotoxicity, we provide a comprehensive and attractive platform for safety testing of API.

## Materials and Methods

### Human Cardiomyocyte Differentiation and Cultivation

Differentiation of human embryonic stem cells (hES) (cell line SA002, Cellartis AB, www.cellartis.com) to hCMC was performed as previously described [8]. For the majority of experiments, the hCMC were transferred to the Universität Leipzig. There, the hCMC were dissected and individually transferred to a round bottomed 96-well plate (Art.471–810, Schubert Laborfachhandel, Germany) and maintained in DMEM supplemented with 10% fetal bovine serum, 1 mM GlutaMAX, 1% nonessential amino acids, and 0.2% penicillin-streptomycin (all by Invitrogen, Germany) on a self-developed gyratory shaker in an incubator at 37°C, 5% CO_2_ and 95% humidity. The hCMC were used for experiments within two weeks. All work with the hES-derived hCMC was performed in accordance with the Robert-Koch-Institut (RKI, Berlin) and the ethical committee of the Universität Leipzig.

### Field Potential Measurements

For the field potential recording of hCMC, self-developed MCA were used [18]. Additionally, electrode and circuit pathways were optimized for bioelectronic analysis [21] and a suction hole was incorporated by backside etching technology (ZMN, TU Ilmenau, Germany). For monitoring, all MEA/MCA systems were placed in a heatable MEA1060 amplifier system (Multi Channel Systems, Germany). For the majority of the experiments, the hCMC were placed into the cavities by pipette. For demonstrating the automated positioning, a self-developed hydrodynamic positioning system was used. For the hCMC attachment experiments, microelectrode arrays (MEA) (Multi Channel System, Germany) were used as previously described [16]. For the monolayer experiments, 50 hCMC were dissociated by incubation and gently shaking in 0.25% trypsin/EDTA (Invitrogen, Germany) for 20 minutes followed by mechanical dissociation and reseeding on MEA with platinum electrodes (Ayanda, Swiss). From all electrodes where electrical activity was observed, field potential streams were recorded with a sampling rate of 4 kHz by MC-Rack v3.7 software (Multi Channel Systems, Germany). Data was analyzed and processed offline using a self-developed software FiPA v2 programmed with MATLAB (Mathworks, USA) to detect action potentials (manual threshold setting) and determine field potential-derived action potential duration (fAPD). For all analyses, 100 detected spikes were averaged and for statistical analysis the determined fAPD was corrected according to Fridericia (fAPD_C_). For MCA-based long-term measurements, fAPD was determined as return to baseline with a threshold of three times s.d. of the baseline and the fAPD_C_ of all four electrodes per cavity were averaged. For short-term analysis where the position of the hCMC does not change within the cavity, the electrode with the most distinct field potential shape was selected.

### Electrochemical Impedance Spectroscopy (EIS)

After field potential measurement the MCA and hCMC were transferred to a self-developed impedance measurement system comprising a 60 channel multiplexer board and a high precision impedance analyzer (ISX-3, ScioSpec GmbH, Germany). The impedance spectra were recorded and analyzed as previously described [20], [21]. Briefly, impedance spectra were recorded by applying an alternating voltage of 100 mV in a frequency range from 5 kHz to 5 MHz with equidistant spacing in a logarithmic scale (51 points). The self-developed software IMAT v2.2.5 was used for instrument controlling and data acquisition. The self-developed software IDAT v3.6.5 was used for offline data analysis, including calculation of the relative impedance (|Z|_with cluster_ − |Z|_without cluster_)/|Z|_without cluster_ × 100%) and determination of the relative impedance maximum. From each cavity that comprises four electrodes, six spectra were acquired (each electrode against each other). Then, the relative impedance maxima of all six spectra were averaged for minimizing disturbance caused by positioning shifts and variations as well as hCMC contraction. To allow comparison and statistical analysis, time traces of the relative impedance maxima were normalized to time point 0 h (start of the experiment).

### Immunocytochemistry

For immunocytochemical staining, hCMC were fixed after the experiments with 4% formaldehyde for one hour, transferred to PBS (Invitrogen, Germany) containing 25% sucrose (Merck, USA) stored for at least 24 hours at 4°C and cryodissected with a Leica CM3050S microtome (20 µm sections). Sections were permeabilized and blocked with 3% BSA and 0.1% Triton-PBS for 45 minutes at room temperature (all from Sigma Aldrich). Afterwards, the sections were incubated with anti-MLC-2a, anti-MLC-2v (1∶100, Synaptic Systems, Germany), anti-α-actinin, anti-α-MHC (1∶100, Sigma Aldrich) for two hours followed by incubation with DyLight-488 or DyLight-549 conjugated anti-rabbit or anti-mouse secondary antibodies for 90 minutes (Dianova, Germany). Then, the sections were carefully washed in PBS three times and nuclei were stained by incubation with DAPI (1 µg/ml) for one minute at room temperature in the dark, followed by washing, drying and covering with Kaiser's glycerol gelatine (Merck, USA). Images were taken using a Nikon C1plus confocal microscope (TE2000).

### Cardiac Troponin Release Analysis

For cardiac troponin release experiments, hCMC were excised from culture and re-plated into individual wells of a 96-well plate. The culture medium was replaced by doxorubicin-containing medium, which was then sampled after 48 hours and measured for cardiac specific troponin content using an automated immuno-chemiluminescence instrument (Elecsys 2010, Roche) and the Elecsys TroponinT STAT assay reagent (Roche) according to the manufacturer’s recommendations.

### Statistics

All statistical analyses were done using Graphpad Prism 5. In general, results are presented as means ± s.e.m. unless described differently. All EC_50_ and IC_50_ values were determined by nonlinear regression fit for agonists/antagonists (three parameters) and CI_95_ indicates the range within the 95% confidence interval. One group comparisons were analyzed by one-way ANOVA and Dunnett post hoc test, multiple group comparisons were analyzed by two-way ANOVA and Bonferroni post hoc test. Differences between two means with p<0.05 were considered as statistically significant (*), p<0.01 very significant (**) and p<0.001 extremely significant (***).

## Results

### Characterization of MCA-based hCMC Monitoring System

**Figure 1:**
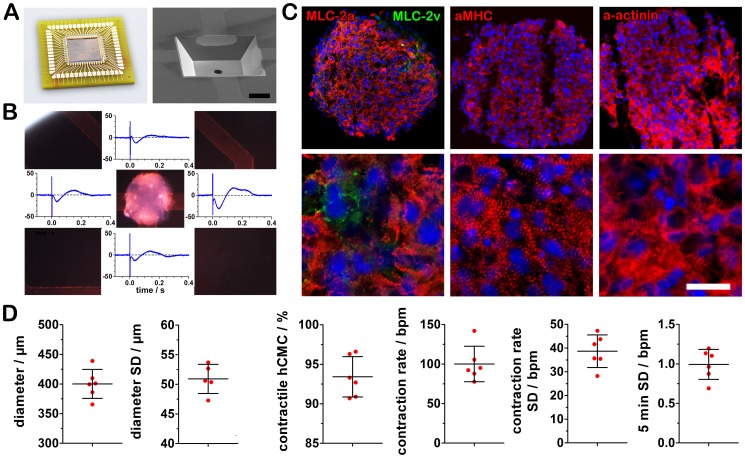
Optimized microcavity array for field potential analysis of highly purified cardiomyocyte clusters. (A) Bonded silicone-based microcavity array and SEM image of a cavity (400 µm size) with an integrated suction hole for automated positioning. (B) Microcavity array technology enables an instant and detailed field potential recording on four electrodes per cluster without any adhesion requirement of the cardiomyocyte cluster to the electrodes. (C) The hCMC consist of highly enriched cardiomyocytes with atrial (MLC-2a) and to a smaller amount of ventricular cardiomyocyte characteristics (MLC-2v), (bar  = 100 µm, zoom: bar  = 20 µm). (D) Size and electrophysiological characteristics of six independent differentiation experiments each with 36 hCMC (mean ± s.d.).

The MCA was developed and optimized for instant and feasible bioelectronic monitoring of human stem cell-derived hCMC (Figure 1A). The silicone-based chip comprises 15 cavities with 200–400 µm size in square (50 µm steps). A gold electrode is located on each cavity wall and a suction hole was incorporated at the cavity bottom giving the possibility for an automated hydrodynamic positioning of the hCMC (Movie S1). This unique three-dimensional analytic structure allows the detection of the action potential by field potential measurement. More strikingly, the optimized electrode size and shape in combination with the gravity-based contacting of the hCMC to the electrode results in the field potential-derived detection of the action potential duration (fAPD) on all four electrodes per cavity (Figure 1B). In contrast to planar MEA, where fAPD detection is strictly dependent on the adhesion quality of the cells, the MCA does not require cell adhesion to the electrode. Furthermore, adhesion of the cluster is prohibited by a repellent silicon nitride passivation layer, making retrieval of the hCMC as well as long-term measurements possible without alteration of the cluster structure and shape (Movie S2). Before experiments with hCMC were performed, they were characterized by immunocytochemical stainings. The analysis of the cardiomyocyte markers α-actinin, α-MHC and subtype marker MLC-2v (ventricular) and MLC-2a (atrial) revealed a high percentage of cardiomyocytes within the clusters (Figure 1C). To investigate the potential presence of non-cardiomyocytes, stainings for the pluripotency marker Oct4, the fibroblast marker CD90, and for other mesodermal origin the marker vimentin were performed (Figure S1). Beside some negligible non-cardiomyocyte cells, characterized by the vimentin staining, the analysis confirmed the high enrichment of cardiomyocytes within the hCMC and is in line with previous characterization of this type of hCMC [8], [9]. For demonstration of high reproducible size and electrophysiological characteristics, we analyzed six independent differentiation experiments each with 36 hCMCs (Figure 1D). The hCMCs revealed a highly reproducible diameter with an average of 400.2±25.5 µm and an intra-experiment s.d. of 50.9±2.5 µm in average. Moreover, 93.4±2.6% of the hCMCs were contractile and showed a highly reproducible contraction rate of 100.1±22.5 bpm in average and an intra-experiment s.d. 38.7 4±6.9 bpm.

### MCA Technology Provides High Signal Quality Enabling Feasible Long-term Monitoring of hCMC

**Figure 2:**
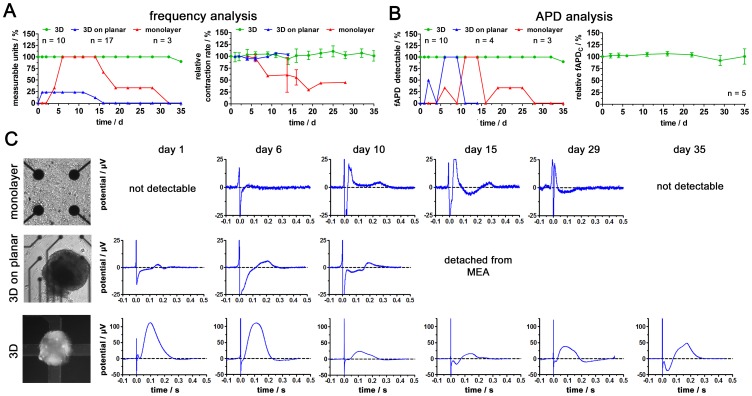
MCA technology improves quality and long-term stability of field potential-derived action potential parameters. Comparative analysis of available techniques (monolayer cultures and hCMC attached to planar MEA) and the MCA technology. (A) Analysis of contractility and contraction rate stability for each method over 35 days. (B) Analysis of fAPD occurrence and fAPD stability for each method over 35 days. (C) Representative examples of recorded field potentials from the three methods.

With the intention to use the established hCMC based bioelectronic monitoring system for long-term experiments, we analyzed the size and morphological stability of the hCMC over 35 days (Figure S2). Both microscopic images as well as the hCMC size analysis showed no significant alterations. To demonstrate the superior signal quality and long-term stability of MCA-based field potential recording on hCMC, this novel technology was compared to the commonly used techniques of dissociated and reseeded cardiomyocytes on planar MEA and hCMC attached to planar MEA. Firstly, the cardiomyocyte contraction rates measured in the three systems were analyzed (Figure 2A and Table S1). In total, the MCA group (3D) contained ten hCMC and the 3D on planar MEA 17 hCMC. For the monolayer group, 50 hCMC were enzymatically dissociated and reseeded on the planar MEA. While contraction rates could be detected from all ten hCMC in the MCA from day 0 until day 32 (nine hCMC on day 35), only four of the 17 hCMC could be attached to the planar MEA. These four hCMC could be monitored from day 1 until day 11 (two hCMC until day 14). Afterwards, they detached from the MEA. In the case of the monolayer group, it was only possible to record contractions after four days of cultivation on the MEA and only one MEA could be monitored up to day 28. Moreover, the stability of the contraction rate over the monitored days was analyzed. While the four attached hCMC (3D on planar) showed high contraction rate stability with 103.8% at day 14 when compared to day 1 (100%) and a low variance with 4.6% (s.d.) in average, the monolayer system showed a rapid decrease of contraction rate down to 55.6% at day 16 and 45.0% on day 29 when compared to day 4/6. In addition, the variance increased to more than 15% (s. d.) between day 6 and 19. Notably, the MCA-based 3D system showed extremely stable contraction rates until day 35 with 100.8% (in comparison relative to day 0) and an averaged variance of 9.0% (s.d.). Moreover, the statistical analysis revealed that these deviations are not significant.

Beside contraction rate analysis, the fAPD determination is another important parameter for cardiotoxicity testing. Therefore, the ability to detect fAPD was analyzed in all three systems (Figure 2B). While for the 3D on planar MEA system fAPD could be determined between day 2 and 9, in the monolayer system it could be determined from day 6 to 25. More strikingly, in the MCA-based 3D system, fAPD could be determined in all contractile hCMC over the completely monitoring period of 35 days. For demonstrating the long-term stability of the fAPD, five hCMC were analyzed over the whole monitoring time range showing high stability and low variances with 106.1% at day 35 when compared to day 0 (100%). Moreover, the statistical analysis revealed that these deviations are not significant. To illustrate alterations in field potential profile quality over time, example electrodes from each system are shown in Figure 2C. The most suitable time for fAPD experiments for the monolayer system was between day 10 and 15, while for the 3D on planar system this was between day 6 and 13. As indicated above, the MCA-based measurements on hCMC showed an extremely distinct field potential shape over the whole monitoring time range of 35 days.

### MCA-based Electrophysiological Analysis of hCMC Shows High Sensitivity to Compound Effects

**Figure 3:**
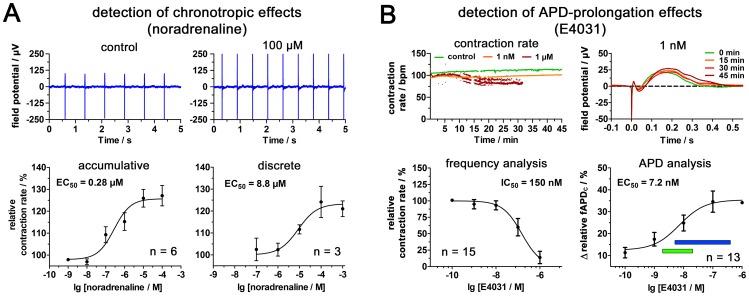
hCMC for real-time detection of API adverse effects. (A) The recorded field potential of one hCMC at the beginning (control) and after application of 100 µM noradrenaline. The comparison of the concentration response curve for the accumulative (n  = 6 hCMC) and discrete application (n  = 3 hCMC) of noradrenaline reveals considerably different EC_50_ values. (B) Analysis of adverse side effects like QT interval relevant APD prolongation can be detected and quantified. While E4031 has an influence on contraction rate at higher concentrations, the application of 1 nM results in a prolonged fAPD after 15 minutes. The analysis of the frequency-corrected fAPD (fAPD_c_) revealed an EC_50_ of 7.2 nM (n  = 13 hCMC), (blue bar – published EC_50_ range obtained from hERG-channel assay, green bar – published EC_10_ range of *in vivo* studies in dogs, monkeys and humans).

After confirmation of the quality and feasibility of the MCA-based field potential analysis, hCMC sensitivity to chronotropic compounds and compounds with adverse effects on the APD were investigated. The hCMC were monitored before and after application of noradrenaline (Figure 3A and Table S2). The recorded field potential before and after the application of 100 µM noradrenaline revealed a considerable increase in contraction frequency. A concentration response curve obtained by the commonly used mode of accumulative application indicated an EC_50_ value of 0.28 µM (CI_95_∶0.075 µM –1.0 µM). A comparative experiment, however, where the different concentrations were applied to a discrete set of hCMC resulted in an EC_50_ value of 8.8 µM (CI_95_∶1.8 µM –43 µM). In a next step, the capability of the MCA-based 3D system to detect APD prolongation by field potential monitoring was evaluated (Figure 3B and Table S2). E4031 is known to induce QT-prolongation and TdP by APD prolongation. Furthermore, as previous animal studies revealed comparable results to human studies (EC_10_ values in the range of 1–20 nM) this is a suitable reference compound for characterizing novel test systems [23]. While E4031 concentrations in the nanomolar range had no considerable influence on the contraction rate, concentrations in the micromolar range caused higher variances, arrhythmias and loss of contraction after 30 minutes of incubation. The prolongation of the fAPD shown for 1 nM E4031 revealed considerable prolongation after 15–30 minutes with a maximum after 45 minutes. The concentration response analysis revealed an IC_50_ value of 150 nM (CI_95_∶82 nM –270 nM) for the frequency and an EC_50_ value of 7.2 nM (CI_95_∶1.3 nM –40 nM) for the frequency corrected APD prolongation. This value is in line with a previous study on ES-derived human cardiomyocytes [16].

### Multi-parametric Label-free Analysis of Adverse Effects Reveals Comprehensive Information on Induced Cardiotoxicity

**Figure 4:**
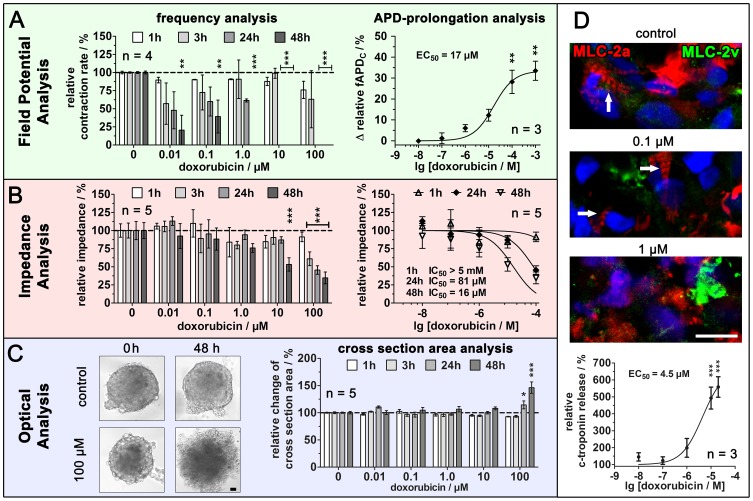
Label-free real-time multi-parametric monitoring enables high content analysis. (A) The contraction rate was analyzed for 48 hours (n  = 4 hCMC). The effect on the APD was determined for one hour after doxorubicin application and revealed a concentration dependent response with an EC_50_ value of 17 µM (n  = 3 hCMC), (B) The impedance analysis revealed a concentration- and time-dependent decrease where IC_50_ values could be determined for each time point (n  = 5 hCMC). (C) The microscopic analysis of the hCMC size (cross section area) showed an increase only for 100 µM after 24 hours and 48 hours (n  = 5). (D) After the experiment, the clusters were suitable for further molecular biological analysis. The immunocytochemical staining indicated typical stratification of contractile elements (arrows) in the control and 0.1 µM treated sample. These stratified elements were not observed in samples treated with 1 µM doxorubicin (bar  = 50 µm). For comparison with established *in vivo* cardiomyocyte damage markers, a c-troponin assay was performed on the culture media.

The capability of direct and repeated measurement of hCMC in the MCA enables long-term monitoring of compounds with regard to their potential cardiotoxic effects together with multiplexing of several label-free and non-invasive analysis methods. In this context, field potential monitoring in combination with impedance spectroscopy analysis was performed. Moreover, hCMC morphology was analyzed by light microscopy. The performance and advantages of the multi-parametric analysis were demonstrated by monitoring the adverse effects of doxorubicin over 48 hours. Effects on the contraction rate were observed by analyzing the field potential (Figure 4A and Table S3). When compared to the time point prior to doxorubicin application, no considerable changes were observed within the first hour for the concentration range of 0.01–10 µM. At 100 µM, a decrease to 76±12% was observed. In contrast, after three hours and up to 48 hours a concentration and time dependent decrease could be observed, even at the lowest concentration of 0.01 µM. In particular, at higher concentrations of 10 µM and 100 µM the hCMC stopped beating after 24 hours. Significant doxorubicin-mediated effects were also observed on the fAPD within the first hour of application (1 mM, 33.5±4.5% increase relative to pre-exposure values). The concentration response analysis revealed an EC_50_ value of 16.7 µM (CI_95_∶5.4 µM –52.0 µM). Additional information on cell-cell contact integrity, as well as cell and cytoskeleton degradation within the 3D-cultures was obtained through impedance spectroscopy [18], [24], [25] analysis (Fig 4B and Table S4). After one hour of incubation with doxorubicin, no obvious alterations could be observed. After three hours, at least for 100 µM doxorubicin, the relative impedance decreased significantly down to 61±9%. After 48 hours, a significant decrease down to 53±9% could also be detected at 10 µM doxorubicin. The IC_50_ values, determined from concentration response curves, were >5 mM for one hour, 150 µM (CI_95_∶71 µM –310 µM) for three hours, 81 µM (CI_95_∶47 µM –142 µM) for 24 hours, and 16 µM (CI_95_∶6 µM –42 µM) for 48 hours.

Changes in the individual cross section area of each hCMC at each time point were evaluated from light microscopy images (Figure 4C and Table S5). Only the highest concentration of doxorubicin revealed a significant alteration (increases to 115±7% and 146±11% after 24 and 48 hours respectively, compared to pre-exposure values). Based on the acquired images, the increased diameter could be correlated with a loosening of the cell-cell contacts and structure resulting in an increased extracellular space and degradation of the hCMC.

Taken together, the results of all three label-free analysis methods demonstrated that doxorubicin exposure led to a fAPD-prolongation within the first hour and considerably influenced the contraction rate within three hours. Degeneration of cells and cell-cell contacts were time- and concentration-dependent, and were already observed after three hours at high concentrations (≥100 µM). Finally, at the highest concentration (100 µM) the cellular degeneration leads to a structural alteration in the whole hCMC with loosened structures and increased cluster size.

To correlate the observed degenerative processes with subcellular changes in the cytoskeleton, we performed immunocytochemical stainings (Figure 4D) of the exposed hCMC after the final time point of the experiments (48 hours). The analysis of the ventricular and especially the atrial marker MLC-2 revealed stratified structures that are part of the contractile fibers within cardiomyocytes in the control hCMC and in hCMC incubated with up to 0.1 µM doxorubicin. At concentrations of 1 µM and higher, no stratified structures were observed, which is in line with the complete loss of contractility of all clusters in this concentration range. In addition, the stainings showed more dense and aggregated areas of MLC-2 that correlated with the degeneration of the cells at concentrations higher than 1 µM doxorubicin. To be able to relate these results to established *in vivo* findings, the translational safety biomarker for drug induced toxicity [26], cardiac specific troponin, was measured in the cell culture medium after 48 hours exposure to doxorubicin (Table S6). Cardiac troponin release was concentration dependent (Figure 4D), and showed a significant increase of c-troponin release at 10 µM doxorubicin and higher with an EC_50_ value of 4.5 µM (CI_95_∶1.1 µM –17.5 µM) that correlates with the performed impedance spectroscopy analysis.

## Discussion

Facing the great opportunity that stem cell derived hCMC offers for the safety assessment of cardiotoxic adverse effects of novel API, there is an urgent demand for analytical methods of these three-dimensional cultures that can be easily upscaled and multiplexed. Moreover, long term monitoring of whole hCMC over days or weeks is desired especially in the context of chronic toxicity testing. Therefore, three-dimensional cultures consisting of highly enriched cardiomyocytes as demonstrated by the immunocytochemical staining are a prerequisite. While α-MHC and α-actinin are common markers for cardiomyocytes, MLC-2a and MLC-2v are specific markers for cardiac subtypes. Moreover, these markers plays a crucial role during maturation of cardiomyocytes making it difficult for definite cardiac subtype identification. Actual studies revealed that MLC-2v positive cells represents more matured ventricular cardiomyocytes while MLC-2a only positive cells could represent unmatured cardiomyocytes and therefore, a range of potential cardiomyocyte subtypes including ventricular as well as atrial cardiomyocytes [27]–[29].

To overcome the limitations for label-free field potential monitoring of hCMC on planar MEA, we developed a unique MCA. The major advantage of the MCA-based system is that for optimum signal quality, adhesion of the hCMC to the electrodes is not needed and therefore, greatly simplifies the practical aspects of the system. Several studies of planar MEA systems have demonstrated the importance of the development of an optimum cell adhesion on the electrodes over several days to obtain a good signal quality, especially for the detection and determination of the fAPD [15], [30]. In contrast, we were able to demonstrate for the first time, that by using the MCA, the gravity-based contact between electrode and hCMC is sufficient to obtain a pronounced field potential. More strikingly, the fAPD could be feasibly determined. This allows an easy and fast processing as well as measurement of hCMC. Furthermore, the repeated measurements of individual hCMC over days or even weeks can be performed without the need of any disruption, digestion, or plating typically causing deformation of the 3D structure that developed during differentiation. The integrity of cluster structure/shape was demonstrated by experiments over eight hours of continuous measurements as well as discrete measurements over 35 days. During this time, hCMC showed superior stability and microscopic characterization never revealed degeneration within the hCMC. This is in line with previous studies [8], [9]. The comparative analysis of long-term stability in monolayer cultures on MEA (monolayer), attached hCMC on MEA (3D on planar) and hCMC on the MCA-based system (3D) revealed superior advantages with regard to contractile clusters/layers, contraction rate variances, and detection of fAPD. Only with the MCA-based system, we could demonstrate, for the first time, the stable long-term (35 days) tracing of the fAPD in individual hCMC. In the context of automation for industrial application, the manual preparation of the hCMC at the beginning is a limiting step. However, the ongoing development of stem cell culturing in 3D as well as the differentiation to hCMC under well-defined automatable conditions are promising for the industrial application of our system [7], [31]. Although the well-established hES-derived hCMC show superior functional characteristics over more than 35 days, the MCA based measurement system is not restricted to hES-derived hCMC. Induced pluripotent stem cells (hiPS) offers a great opportunity for the generation of hCMC and therefore, the MCA based system are highly suitable for the functional investigation of hiPS-derived hCMC. Moreover, patient derived hiPS allows the establishment of specific hCMC pathology models that could be used for sophisticated screening systems [32].

Using the MCA in combination with the hCMC, we were able to demonstrate the feasibility of our system to detect and quantify short-term effects of frequency modulating compounds and APD prolonging compounds. For the chronotropic acting noradrenaline, we demonstrated accumulative as well as discrete EC_50_ determination with a considerable higher value in the discrete measurement, but both in the range of formerly reported EC_50_ values of 1.7 µM [33]. Although, accumulative experiments are widely used because of lower experimental variances, lower material consumption and time, for most targets there is little known about, for example desensitizing and/or receptor internalization. We show in the present study, that comparable CI_95_ ranges were achieved in both experimental settings using the MCA-based system. Thus, parallel measurements on individual hCMC and discrete analysis of compound effects are now more easily achievable, avoiding the confounding factors possibly introduced by an accumulative experimental set-up.

The fAPD-prolongation analysis of E4031 revealed an EC_50_ value that is in line with former described studies on hCMC [16]. Since E4031 is a hERG channel blocker, the determined EC_50_ value is comparable with reported values obtained by hERG channel assays [23]. Moreover, at higher concentrations we could observe adverse effects on the contraction rate and overall contractility. This can be caused by nonspecific action on different channels [16].

Especially for long-term experiments that increase experimental time and efforts, we demonstrated the advantages of multi-parametric label-free monitoring of individual hCMC. For this purpose, the effects of doxorubicin were analyzed by field potential recording, impedance spectroscopy, and microscopic imaging. Within 1 hour after doxorubicin application, we could detect a fAPD-prolongation (EC_50_ = 18 µM). Since doxorubicin acts over an I_Kr_ independent mechanism, it is not a classical reference compound for QT prolongation and TdP [34]. Only in the recent literature there are indications of the arrhythmic adverse effects of doxorubicin [35]. This shows the high predictive power of our MCA-based system in combination with hCMC. Moreover, the long-term effects related to structural cardiotoxicity could be sensitively monitored by alterations of the contraction rate. Here, we observed a significant decrease of the contraction rate already at 0.1 µM doxorubicin after 48 days. Furthermore, our system is much more sensitive than actual published hES derived cardiomyocyte monitoring systems that, thus far, never revealed an effect of doxorubicin on contractility at concentrations of 100 µM and higher [36]. In combination with a concentration- and time-dependent decrease of the relative impedance, the degradation of the cytoskeleton was confirmed with immunocytochemical staining’s. The sensitivity of the multi-parametric analysis was further investigated by measuring release of the clinically established biomarker cardiac troponin. The determined EC_50_ value (4.5 µM) is in line with previous studies [37]. The comparable results obtained by the bioelectronic multi-parametric analysis illustrate the direct relevance to human health and provide connectivity to *in vivo* results. More strikingly, we could detect significant effects of doxorubicin in the concentration range of 0.1 µM –20 µM doxorubicin that represents in vivo relevant concentrations and that are lower than already described C_max_ values of 10–35 µM of doxorubicin [36], [38]. Using the MCA technology, the electrophysiological properties of the cardiomyocytes can be assayed in combination with measurements of cell viability. Hence, general cytotoxicity can be distinguished from specific cardiotoxicity.

### Conclusions

Taken together, the MCA-based multi-parametric non-invasive and label-free bioelectronic and optical monitoring of individual hCMC provides a superior tool for the reliable long-term monitoring of hCMC and detailed analysis of adverse cardiotoxic effects especially in the context of long-term chronic and repeated dose toxicity testing of potential API. Especially in the context of accumulative effects over days and weeks as shown for doxorubicin [35] there is a need of novel highly predictable *in vitro* test systems. The demonstrated hES-derived hCMC in combination with the MCA-based measurement system can be easily upscaled to e.g. standard 96-well plates leading to a high-content analysis platform and would push stem cell derived hCMC forward to reliable long-term *in vitro* cardiotoxicity testing. Moreover, the developed system is not limited to the use of hES-derived hCMC but presents a suitable technique for the characterization and investigation of hiPS-derived hCMC.

## Acknowledgments

The research leading to these results has received funding from the European Community's Seventh Framework Program ([FP7/2007–2013]) and Cosmetics Europe under Grant Agreement No.266753 (SCR&Tox) as well as by the German Federal Ministry of Education and Research (BMBF, Project: BiMiHySS FKZ 16SV5052). The image acquisition facility (confocal microscope) was funded by the Free State of Saxony and the European Union (SMWK/EFRE).

## Supporting Information

Figure S1 **Immunocytochemical characterization of hCMC for non-cardiomyocyte cells.** The hCMC cryosections were stained for vimentin as mesodermal-derived cells that are non-cardiomyocytes, CD90 as a fibroblast marker and Oct3/4 as a stem cell marker. Adult human dermal fibroblast (HDFa) and murine embryonic stem cells (ES-D3) were used as positive controls. (bar  = 100 µm).
(TIF)

Figure S2 **Long-term stability of hCMCs.** Ten hCMCs were monitored by microscopy over 35 days. The microscopy images revealed no significant alterations (exemplarily shown for one hCMC). Moreover, the diameter showed no significant changes over the whole 35 days (mean ± s.d.). (bar  = 200 µm).
(TIF)

Table S1 **Quantitative analysis of field potential parameter long-term stability.** (mean ± s.e.m, (n)).
(DOCX)

Table S2 **Quantitative field potential parameter analysis of noradrenaline- and E4031-treated hCMC.** (mean ± s.e.m).
(DOCX)

Table S3 **Quantitative field potential parameter analysis of doxorubicin-treated hCMC.** (mean ± s.e.m).
(DOCX)

Table S4 **Quantitative impedance spectroscopy analysis of doxorubicin-treated hCMC.** (mean ± s.e.m).
(DOCX)

Table S5 **Quantitative cross section area analysis of doxorubicin-treated hCMC.** (mean ± s.e.m).
(DOCX)

Table S6 **Quantitative c-tropnonin release analysis of doxorubicin-treated hCMC.** (mean ± s.e.m).
(DOCX)

Movie S1 **Automated hydrodynamic positioning of a hCMC.**
(MPG)

Movie S2 **Non-adhered beating hCMC positioned in a microcavity.**
(AVI)

## References

[1] Mandenius CF, Steel D, Noor F, Meyer T, Heinzle E, et al (2011) Cardiotoxicity testing using pluripotent stem cell-derived human cardiomyocytes and state-of-the-art bioanalytics: a review. J Appl Toxicol 31: 191–205. 21328588 10.1002/jat.1663

[2] Jonsson MK, Vos MA, Mirams GR, Duker G, Sartipy P, et al. (2012) Application of human stem cell-derived cardiomyocytes in safety pharmacology requires caution beyond hERG. J Mol Cell Cardiol. 10.1016/j.yjmcc.2012.02.002 22353256

[3] Sheppard RJ, Berger J, Sebag IA (2013) Cardiotoxicity of cancer therapeutics: current issues in screening, prevention, and therapy. Front Pharmacol 4: 19. 23487556 10.3389/fphar.2013.00019 PMC3594741

[4] Lu HR, Marien R, Saels A, De Clerck F (2001) Species plays an important role in drug-induced prolongation of action potential duration and early afterdepolarizations in isolated Purkinje fibers. J Cardiovasc Electrophysiol 12: 93–102. 11204092 10.1046/j.1540-8167.2001.00093.x

[5] Asp J, Steel D, Jonsson M, Ameen C, Dahlenborg K, et al (2010) Cardiomyocyte clusters derived from human embryonic stem cells share similarities with human heart tissue. J Mol Cell Biol 2: 276–283. 20802012 10.1093/jmcb/mjq022

[6] Ye L, Zhang S, Greder L, Dutton J, Keirstead SA, et al (2013) Effective cardiac myocyte differentiation of human induced pluripotent stem cells requires VEGF. PLoS One 8: e53764. 23326500 10.1371/journal.pone.0053764 PMC3542360

[7] Burridge PW, Thompson S, Millrod MA, Weinberg S, Yuan X, et al (2011) A universal system for highly efficient cardiac differentiation of human induced pluripotent stem cells that eliminates interline variability. PLoS One 6: e18293. 21494607 10.1371/journal.pone.0018293 PMC3072973

[8] Synnergren J, Akesson K, Dahlenborg K, Vidarsson H, Ameen C, et al (2008) Molecular signature of cardiomyocyte clusters derived from human embryonic stem cells. Stem Cells 26: 1831–1840. 18436862 10.1634/stemcells.2007-1033

[9] Synnergren J, Ameen C, Jansson A, Sartipy P (2012) Global transcriptional profiling reveals similarities and differences between human stem cell-derived cardiomyocyte clusters and heart tissue. Physiol Genomics 44: 245–258. 22166955 10.1152/physiolgenomics.00118.2011

[10] Steel D, Hyllner J, Sartipy P (2009) Cardiomyocytes derived from human embryonic stem cells - characteristics and utility for drug discovery. Curr Opin Drug Discov Devel 12: 133–140. 19152222

[11] Zhu WZ, Santana LF, Laflamme MA (2009) Local control of excitation-contraction coupling in human embryonic stem cell-derived cardiomyocytes. PLoS One 4: e5407. 19404384 10.1371/journal.pone.0005407 PMC2671137

[12] Vidarsson H, Hyllner J, Sartipy P (2010) Differentiation of human embryonic stem cells to cardiomyocytes for in vitro and in vivo applications. Stem Cell Rev 6: 108–120. 20091143 10.1007/s12015-010-9113-x

[13] Nalos L, Varkevisser R, Jonsson MK, Houtman MJ, Beekman JD, et al (2012) Comparison of the IKr blockers moxifloxacin, dofetilide and E-4031 in five screening models of pro-arrhythmia reveals lack of specificity of isolated cardiomyocytes. Br J Pharmacol 165: 467–478. 21718297 10.1111/j.1476-5381.2011.01558.x PMC3268199

[14] Otsuji TG, Minami I, Kurose Y, Yamauchi K, Tada M, et al (2010) Progressive maturation in contracting cardiomyocytes derived from human embryonic stem cells: Qualitative effects on electrophysiological responses to drugs. Stem Cell Res 4: 201–213. 20199896 10.1016/j.scr.2010.01.002

[15] Rothermel A, Kurz R, Ruffer M, Weigel W, Jahnke HG, et al (2005) Cells on a chip–the use of electric properties for highly sensitive monitoring of blood-derived factors involved in angiotensin II type 1 receptor signalling. Cell Physiol Biochem 16: 51–58. 16121033 10.1159/000087731

[16] Braam SR, Tertoolen L, van de Stolpe A, Meyer T, Passier R, et al (2010) Prediction of drug-induced cardiotoxicity using human embryonic stem cell-derived cardiomyocytes. Stem Cell Res 4: 107–116. 20034863 10.1016/j.scr.2009.11.004

[17] Reppel M, Igelmund P, Egert U, Juchelka F, Hescheler J, et al (2007) Effect of cardioactive drugs on action potential generation and propagation in embryonic stem cell-derived cardiomyocytes. Cell Physiol Biochem 19: 213–224. 17495462 10.1159/000100628

[18] Kloss D, Kurz R, Jahnke HG, Fischer M, Rothermel A, et al (2008) Microcavity array (MCA)-based biosensor chip for functional drug screening of 3D tissue models. Biosens Bioelectron 23: 1473–1480. 18289841 10.1016/j.bios.2008.01.003

[19] Krinke D, Jahnke HG, Panke O, Robitzki AA (2009) A microelectrode-based sensor for label-free in vitro detection of ischemic effects on cardiomyocytes. Biosens Bioelectron 24: 2798–2803. 19285854 10.1016/j.bios.2009.02.006

[20] Haas S, Jahnke HG, Glass M, Azendorf R, Schmidt S, et al (2010) Real-time monitoring of relaxation and contractility of smooth muscle cells on a novel biohybrid chip. Lab Chip 10: 2965–2971. 20835426 10.1039/c0lc00008f

[21] Krinke D, Jahnke HG, Mack TG, Hirche A, Striggow F, et al (2010) A novel organotypic tauopathy model on a new microcavity chip for bioelectronic label-free and real time monitoring. Biosens Bioelectron 26: 162–168. 20591644 10.1016/j.bios.2010.06.002

[22] Jahnke HG, Braesigk A, Mack TG, Ponick S, Striggow F, et al (2012) Impedance spectroscopy based measurement system for quantitative and label-free real-time monitoring of tauopathy in hippocampal slice cultures. Biosens Bioelectron 32: 250–258. 22221799 10.1016/j.bios.2011.12.026

[23] Omata T, Kasai C, Hashimoto M, Hombo T, Yamamoto K (2005) QT PRODACT: comparison of non-clinical studies for drug-induced delay in ventricular repolarization and their role in safety evaluation in humans. J Pharmacol Sci 99: 531–541. 16493193 10.1254/jphs.qt-c12

[24] Kloss D, Fischer M, Rothermel A, Simon JC, Robitzki AA (2008) Drug testing on 3D in vitro tissues trapped on a microcavity chip. Lab Chip 8: 879–884. 18497906 10.1039/b800394g

[25] Jahnke HG, Rothermel A, Sternberger I, Mack TG, Kurz RG, et al (2009) An impedimetric microelectrode-based array sensor for label-free detection of tau hyperphosphorylation in human cells. Lab Chip 9: 1422–1428. 19417909 10.1039/b819754g

[26] Berridge BR, Pettit S, Walker DB, Jaffe AS, Schultze AE, et al (2009) A translational approach to detecting drug-induced cardiac injury with cardiac troponins: consensus and recommendations from the Cardiac Troponins Biomarker Working Group of the Health and Environmental Sciences Institute. Am Heart J 158: 21–29. 19540388 10.1016/j.ahj.2009.04.020

[27] Lee P, Klos M, Bollensdorff C, Hou L, Ewart P, et al (2012) Simultaneous voltage and calcium mapping of genetically purified human induced pluripotent stem cell-derived cardiac myocyte monolayers. Circ Res 110: 1556–1563. 22570367 10.1161/CIRCRESAHA.111.262535 PMC3423450

[28] Kamakura T, Makiyama T, Sasaki K, Yoshida Y, Wuriyanghai Y, et al (2013) Ultrastructural maturation of human-induced pluripotent stem cell-derived cardiomyocytes in a long-term culture. Circ J 77: 1307–1314. 23400258 10.1253/circj.cj-12-0987

[29] Zhang J, Klos M, Wilson GF, Herman AM, Lian X, et al (2012) Extracellular matrix promotes highly efficient cardiac differentiation of human pluripotent stem cells: the matrix sandwich method. Circ Res 111: 1125–1136. 22912385 10.1161/CIRCRESAHA.112.273144 PMC3482164

[30] Halbach M, Egert U, Hescheler J, Banach K (2003) Estimation of action potential changes from field potential recordings in multicellular mouse cardiac myocyte cultures. Cell Physiol Biochem 13: 271–284. 14586171 10.1159/000074542

[31] Ungrin MD, Joshi C, Nica A, Bauwens C, Zandstra PW (2008) Reproducible, ultra high-throughput formation of multicellular organization from single cell suspension-derived human embryonic stem cell aggregates. PLoS One 3: e1565. 18270562 10.1371/journal.pone.0001565 PMC2215775

[32] Narsinh K, Narsinh KH, Wu JC (2011) Derivation of human induced pluripotent stem cells for cardiovascular disease modeling. Circ Res 108: 1146–1156. 21527744 10.1161/CIRCRESAHA.111.240374 PMC3098466

[33] Bohm M, La Rosee K, Schwinger RH, Erdmann E (1995) Evidence for reduction of norepinephrine uptake sites in the failing human heart. J Am Coll Cardiol 25: 146–153. 7798493 10.1016/0735-1097(94)00353-r

[34] Redfern WS, Carlsson L, Davis AS, Lynch WG, MacKenzie I, et al (2003) Relationships between preclinical cardiac electrophysiology, clinical QT interval prolongation and torsade de pointes for a broad range of drugs: evidence for a provisional safety margin in drug development. Cardiovasc Res 58: 32–45. 12667944 10.1016/s0008-6363(02)00846-5

[35] Guglin M, Aljayeh M, Saiyad S, Ali R, Curtis AB (2009) Introducing a new entity: chemotherapy-induced arrhythmia. Europace 11: 1579–1586. 19801562 10.1093/europace/eup300

[36] Pointon A, Abi-Gerges N, Cross MJ, Sidaway JE (2013) Phenotypic Profiling of Structural Cardiotoxins In Vitro Reveals Dependency on Multiple Mechanisms of Toxicity. Toxicol Sci. 10.1093/toxsci/kft005 23315586

[37] Andersson H, Steel D, Asp J, Dahlenborg K, Jonsson M, et al (2010) Assaying cardiac biomarkers for toxicity testing using biosensing and cardiomyocytes derived from human embryonic stem cells. J Biotechnol 150: 175–181. 20633583 10.1016/j.jbiotec.2010.06.023

[38] Eckes J, Schmah O, Siebers JW, Groh U, Zschiedrich S, et al (2011) Kinetic targeting of pegylated liposomal doxorubicin: a new approach to reduce toxicity during chemotherapy (CARL-trial). BMC Cancer 11: 337. 21816044 10.1186/1471-2407-11-337 PMC3175222

