# Supplementary figures and images for: A Novel 3D Label-Free Monitoring System of hES-Derived Cardiomyocyte Clusters: A Step Forward to In Vitro Cardiotoxicity Testing
Source: PLoS One. 2013 Jul 8;8(7):e68971. doi: 10.1371/journal.pone.0068971 (PMC3704625; doi:10.1371/journal.pone.0068971)

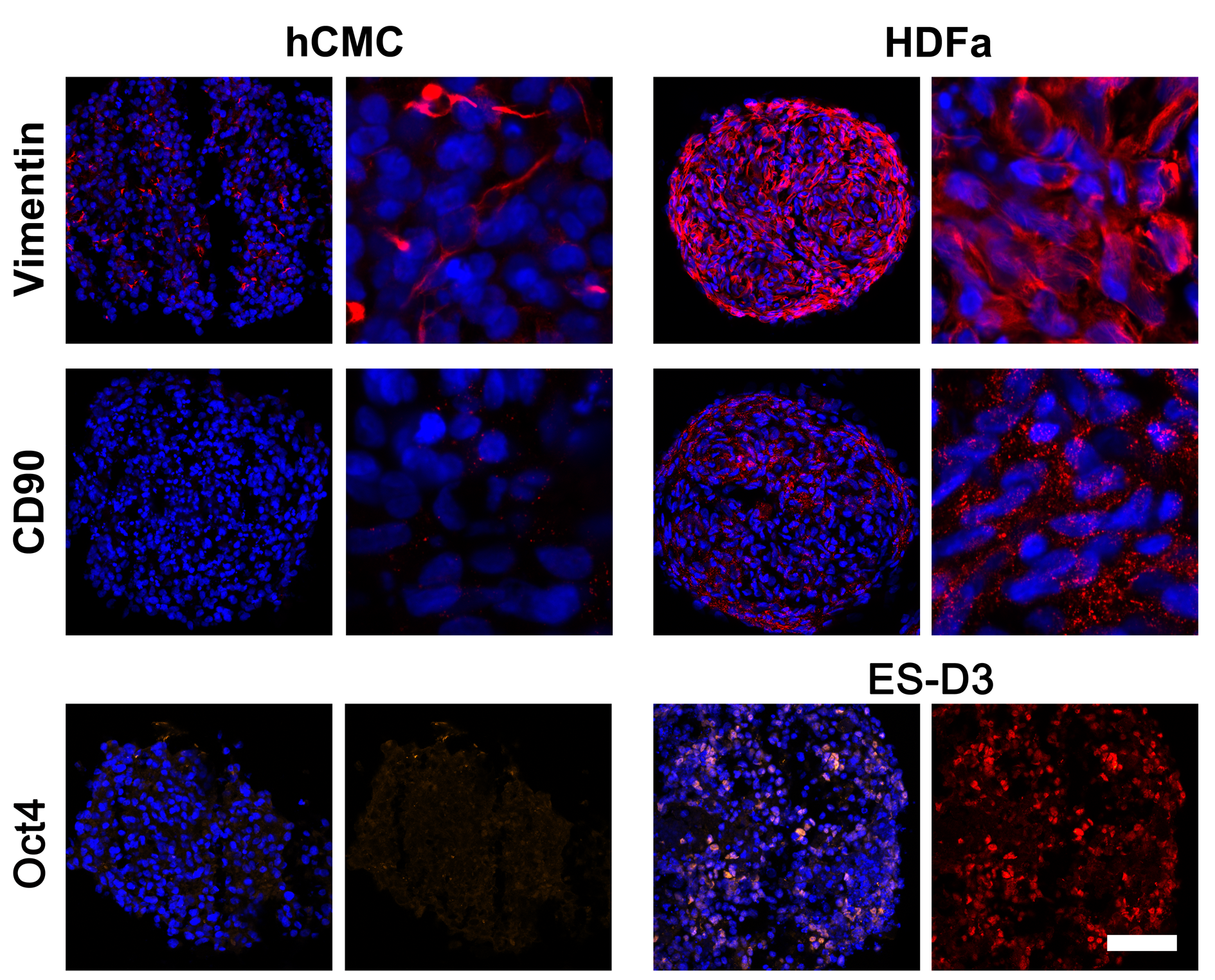

Supplement: Figure S1 — Immunocytochemical characterization of hCMC for non-cardiomyocyte cells. The hCMC cryosections were stained for vimentin as mesodermal-derived cells that are non-cardiomyocytes, CD90 as a fibroblast marker and Oct3/4 as a stem cell marker. Adult human dermal fibroblast (HDFa) and murine embryonic stem cells (ES-D3) were used as positive controls. (bar = 100 µm). (TIF) [file pone.0068971.s001.tif]

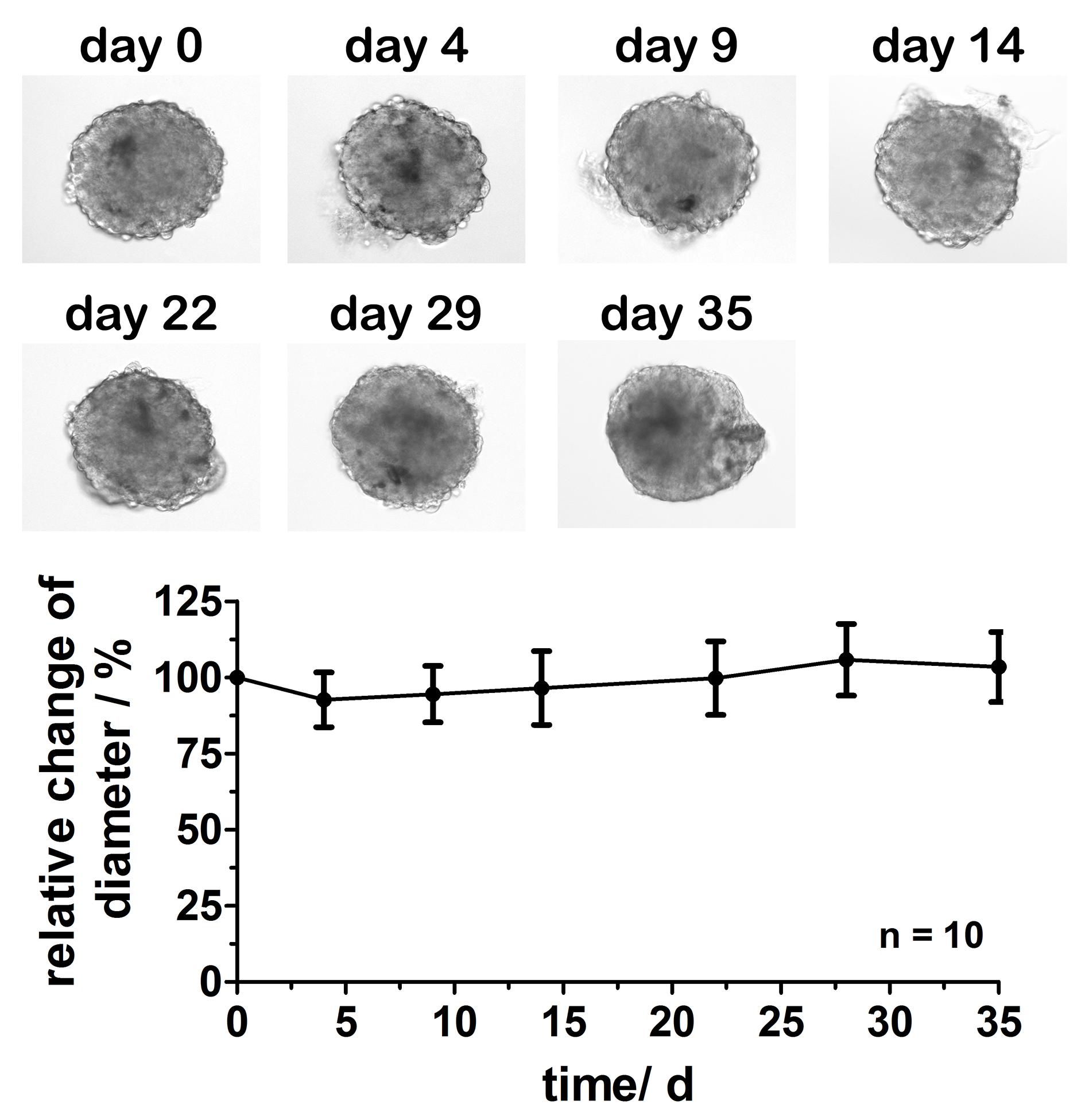

Supplement: Figure S2 — Long-term stability of hCMCs. Ten hCMCs were monitored by microscopy over 35 days. The microscopy images revealed no significant alterations (exemplarily shown for one hCMC). Moreover, the diameter showed no significant changes over the whole 35 days (mean ± s.d.). (bar = 200 µm). (TIF) [file pone.0068971.s002.tif]
