# Supplementary material for: A Novel 3D Label-Free Monitoring System of hES-Derived Cardiomyocyte Clusters: A Step Forward to In Vitro Cardiotoxicity Testing
Source: PLoS One. 2013 Jul 8;8(7):e68971. doi: 10.1371/journal.pone.0068971 (PMC3704625; doi:10.1371/journal.pone.0068971)
Supplement: Table S1 — Quantitative analysis of field potential parameter long-term stability. (mean ± s.e.m, (n)). (DOCX) [file pone.0068971.s003.docx]

Table S1.

| **Time (d)** | **relative contraction rate (%)** | | |  | **relative fAPD_C_ (%) for 3D** |
| --- | --- | --- | --- | --- | --- |
|  | **3D** | **3D on planar** | **monolayer** |  |  |
| 0 | 100.0 ± 0.0 (10) |  |  |  | 100.0 ± 0.0 (5) |
| 1 | 98.3 ± 3.1 (10) | 100.0 ± 0.0 (4) |  |  | not determined |
| 2 | 98.9 ± 2.6 (10) | 101.0 ± 2.2 (4) |  |  | 101.8 ± 4.8 (5) |
| 4 | 103.0 ± 3.6 (10) | 95.1 ± 3.4 (4) | 100.0 (1) |  | 102.5 ± 4.9 (5) |
| 6 | 104.1 ± 1.6 (10) | 94.6 ± 3.3 (4) | 95.8 ± 4.2 (3) |  | 101.5 ± 2.8 (5) |
| 9 | 104.6 ± 1.5 (10) | 99.1 ± 2.8 (4) | 59.0 ± 1.1 (3) |  | not determined |
| 11 | 102.5 ± 1.7 (10) | 106.6 ± 1.8 (4) | not determined |  | 104.8 ± 4.2 (5) |
| 14 | 94.1 ± 3.6 (10) | 103.8 ± 0.7 (4) | 60.9 ± 21.0 (3) |  | not determined |
| 16 | 101.1 ± 4.5 (10) |  | 55.7 ± 9.3 (3) |  | 106.1 ± 4.7 (5) |
| 19 | 101.7 ± 2.8 (10) |  | 29.6 ± 0.5 (2) |  | not determined |
| 22 | 106.4 ± 2.8 (10) |  | 43.8 (1) |  | 103.8 ± 4.6 (5) |
| 25 | 110.3 ± 3.6 (10) |  | not determined |  | not determined |
| 28 | 102.1 ± 2.3 (10) |  | 45.0 (1) |  | 91.8 ± 10.6 (4) |
| 32 | 106.6 ± 2.8 (10) |  |  |  | not determined |
| 35 | 100.8 ± 3.6 (9) |  |  |  | 100.3 ± 16.0 (4) |
