# Supplementary material for: A Novel 3D Label-Free Monitoring System of hES-Derived Cardiomyocyte Clusters: A Step Forward to In Vitro Cardiotoxicity Testing
Source: PLoS One. 2013 Jul 8;8(7):e68971. doi: 10.1371/journal.pone.0068971 (PMC3704625; doi:10.1371/journal.pone.0068971)
Supplement: Table S2 — Quantitative field potential parameter analysis of noradrenaline- and E4031-treated hCMC. (mean ± s.e.m). (DOCX) [file pone.0068971.s004.docx]

Table S2.

| **concentration (M)** | **noradrenaline** | |  | | | **E4031** | | |
| --- | --- | --- | --- | --- | --- | --- | --- | --- |
|  | **relative contraction rate accumulative (%) (n = 6)** | **relative contraction rate discrete (%) (n = 3)** | |  | **relative contraction rate (%)**  **(n = 15)** | | **∆ relative fAPD_C_**  **(%)**  **(n = 13)** |  |
| 10^-10^ |  |  | |  | 100.9 ± 1.5 | | 11.3 ± 2.4 |  |
| 10^-9^ | 97.9 ± 0.7 |  | |  | 94.9 ± 7.1 | | 17.4 ± 3.1 |  |
| 10^-8^ | 96.9 ± 1.3 |  | |  | 93.2 ± 6.9 | | 24.8 ± 3.6 |  |
| 10^-7^ | 109.3 ± 3.7 | 102.5 ± 5.1 | |  | 59.9 ± 13.2 | | 34.6 ± 4.8 |  |
| 10^-6^ | 115.3 ± 4.0 | 102.4 ± 2.9 | |  | 13.8 ± 9.4 | | 34.1  (n = 1) |  |
| 10^-5^ | 127.1 ± 4.6 | 111.6 ± 2.1 | |  |  | |  |  |
| 10^-4^ |  | 124.1 ± 7.1 | |  |  | |  |  |
| 10^-3^ |  | 121.0 ± 3.6 | |  |  | |  |  |
