# Supplementary material for: A Novel 3D Label-Free Monitoring System of hES-Derived Cardiomyocyte Clusters: A Step Forward to In Vitro Cardiotoxicity Testing
Source: PLoS One. 2013 Jul 8;8(7):e68971. doi: 10.1371/journal.pone.0068971 (PMC3704625; doi:10.1371/journal.pone.0068971)
Supplement: Table S3 — Quantitative field potential parameter analysis of doxorubicin-treated hCMC. (mean ± s.e.m). (DOCX) [file pone.0068971.s005.docx]

Table S3

| **concentration**  **(M)** | **relative contraction rate (%)**  **(n = 4)** | | | |  | **∆ relative fAPD_C_ (%)**  **(n = 3)** |
| --- | --- | --- | --- | --- | --- | --- |
|  | **1 h** | **3 h** | **24 h** | **48 h** |  | **1 h** |
| 0 | 100.0  (± 1.8) | 100.0  (± 1.0) | 100.0  (± 2.0) | 100.0  (± 2.0) |  |  |
| 10^-8^ | 89.6  (± 3.8) | 57.1  (± 28.6) | 47.9  (± 25.7) | 20.5  (± 20.5) |  | -0.1  (n = 1) |
| 10^-7^ | 90.2  (± 0.4) | 72.5  (± 24.2) | 59.9  (± 20.1) | 39.3  (± 22.7) |  | 1.2  (± 2.6) |
| 10^-6^ | 90.7  (± 0.6) | 90.8  (± 26.6) | 61.5  (± 2.1) | 0 |  | 6.1  (± 1.7) |
| 10^-5^ | 87.7  (± 5.7) | 99.0  (± 6.9) | 0 | 0 |  | 12.2  (± 2.8) |
| 10^-4^ | 76.0  (± 11.9) | 63.1  (± 39.4) | 0 | 0 |  | 28.2  (± 5.6) |
| 10^-3^ |  |  |  |  |  | 33.5  (± 4.6) |
