# Supplementary material for: A Novel 3D Label-Free Monitoring System of hES-Derived Cardiomyocyte Clusters: A Step Forward to In Vitro Cardiotoxicity Testing
Source: PLoS One. 2013 Jul 8;8(7):e68971. doi: 10.1371/journal.pone.0068971 (PMC3704625; doi:10.1371/journal.pone.0068971)
Supplement: Table S4 — Quantitative impedance spectroscopy analysis of doxorubicin-treated hCMC. (mean ± s.e.m). (DOCX) [file pone.0068971.s006.docx]

Table S4

| **concentration**  **(M)** | **relative impedance (%)**  **(n = 5)** | | | |  |
| --- | --- | --- | --- | --- | --- |
|  | **1 h** | **3 h** | **24 h** | **48 h** |  |
| 0 | 100.0  (± 9.2) | 100.0  (± 10.3) | 100.0  (± 12.5) | 100.0  (± 11.0) |  |
| 10^-8^ | 106.1  (± 4.0) | 105.7  (± 7.3) | 113.1  (± 6.0) | 92.4  (± 17.1) |  |
| 10^-7^ | 109.9  (± 18.9) | 89.1  (± 19.7) | 95.3  (± 19.6) | 88.2  (± 15.3) |  |
| 10^-6^ | 83.9  (± 20.3) | 79.9  (± 5.2) | 94.1  (± 6.6) | 76.0  (± 6.0) |  |
| 10^-5^ | 84.6  (± 8.7) | 90.4  (± 9.3) | 87.1  (± 4.4) | 53.2  (± 9.2) |  |
| 10^-4^ | 91.2  (± 6.6) | 61.1  (± 9.4) | 45.5  (± 5.9) | 34.7  (± 8.2) |  |
