# Supplementary material for: A Novel 3D Label-Free Monitoring System of hES-Derived Cardiomyocyte Clusters: A Step Forward to In Vitro Cardiotoxicity Testing
Source: PLoS One. 2013 Jul 8;8(7):e68971. doi: 10.1371/journal.pone.0068971 (PMC3704625; doi:10.1371/journal.pone.0068971)
Supplement: Table S5 — Quantitative cross section area analysis of doxorubicin-treated hCMC. (mean ± s.e.m). (DOCX) [file pone.0068971.s007.docx]

Table S5

| **concentration**  **(M)** | **relative change of cross section area (%)**  **(n = 5)** | | | |  |
| --- | --- | --- | --- | --- | --- |
|  | **1 h** | **3 h** | **24 h** | **48 h** |  |
| 0 | 100.0  (± 10.6) | 100.0  (± 1.8) | 100.0  (± 1.1) | 100.0  (± 2.2) |  |
| 10^-8^ | 96.8  (± 1.4) | 102.0  (± 1.0) | 110.5  (± 2.0) | 100.3  (± 3.5) |  |
| 10^-7^ | 102.6  (± 3.5) | 96.4  (± 3.1) | 96.6  (± 4.5) | 104.6  (± 5.1) |  |
| 10^-6^ | 96.3  (± 2.5) | 96.5  (± 3.4) | 97.5  (± 2.2) | 106.5  (± 5.0) |  |
| 10^-5^ | 95.1  (± 1.6) | 94.5  (± 1.4) | 100.1  (± 2.1) | 108.0  (± 2.7) |  |
| 10^-4^ | 92.8  (± 0.5) | 93.3  (± 1.7) | 114.5  (± 7.2) | 146.2  (± 10.5) |  |
