# Supplementary material for: A Novel 3D Label-Free Monitoring System of hES-Derived Cardiomyocyte Clusters: A Step Forward to In Vitro Cardiotoxicity Testing
Source: PLoS One. 2013 Jul 8;8(7):e68971. doi: 10.1371/journal.pone.0068971 (PMC3704625; doi:10.1371/journal.pone.0068971)
Supplement: Table S6 — Quantitative c-tropnonin release analysis of doxorubicin-treated hCMC. (mean ± s.e.m). (DOCX) [file pone.0068971.s008.docx]

Table S6

| **concentration**  **(M)** | **relative c‑troponin release / %**  **(n = 3)** |
| --- | --- |
| 0 | 100.0 (± 10.2)  (n = 4) |
| 10^-8^ | 145.4  (± 24.3) |
| 10^-7^ | 123.7  (± 22.3) |
| 10^-6^ | 198.6  (± 55.6) |
| 10^-5^ | 492.8  (± 64.8) |
| 2 x 10^-5^ | 558.4  (± 60.6) |
